# Relationships between dietary rumen-protected lysine and methionine with the lactational performance of dairy cows — A meta-analysis

**DOI:** 10.5713/ab.23.0084

**Published:** 2023-08-22

**Authors:** Agung Irawan, Ahmad Sofyan, Teguh Wahyono, Muhammad Ainsyar Harahap, Andi Febrisiantosa, Awistaros Angger Sakti, Hendra Herdian, Anuraga Jayanegara

**Affiliations:** 1 Vocational School, Universitas Sebelas Maret, Surakarta 57126, Indonesia; 2 Department of Animal and Rangeland Sciences, Oregon State University, Corvallis 97331, OR, USA; 3 Animal Feed and Nutrition Modelling (AFENUE) Research Group, Faculty of Animal Science, IPB University, Bogor 16680, Indonesia; 4 Research Center for Animal Husbandry, National Research and Innovation Agency (BRIN), Cibinong, Bogor 16911, Indonesia; 5 Research Center for Food Technology and Processing, National Research and Innovation Agency (BRIN), Gunungkidul, Daerah Istimewa Yogyakarta 55861, Indonesia; 6 Department of Nutrition and Feed Technology, Faculty of Animal Science, IPB University, Bogor 16680, Indonesia

**Keywords:** Dairy Cows, Meta-analysis, Milk Protein Synthesis, Rumen-protected Lysine

## Abstract

**Objective:**

Our objective was to examine the relationships of supplemental rumen-protected lysine (RPL) or lysine + methionine (RPLM) on lactational performance, plasma amino acids (AA) concentration, and nitrogen use efficiency of lactating dairy cows by using a meta-analysis approach.

**Methods:**

A total of 56 articles comprising 77 experiments with either RPL or RPLM supplementation were selected and analyzed using a mixed model methodology by considering the treatments and other potential covariates as fixed effects and different experiments as random effects.

**Results:**

In early lactating cows, milk yield was linearly increased by RPL (β_1_ = 0.013; p< 0.001) and RPLM (β_1_ = 0.014; p<0.028) but 3.5% fat-corrected milk (FCM) and energy-corrected milk (ECM) (kg/d) was increased by only RPL. RPL and RPLM did not affect dry matter intake (DMI) but positively increased (p<0.05) dairy efficiency (Milk yield/DMI and ECM/DMI). As a percentage, milk fat, protein, and lactose were unchanged by RPL or RPLM but the yield of all components was increased (p<0.05) by feeding RPL while only milk protein was increased by feeding RPLM. Plasma Lys concentration was linearly increased (p<0.05) with increasing supplemental RPL while plasma Met increased (p<0.05) by RPLM supplementation. The increase in plasma Lys had a strong linear relationship (*R*^2^ = 0.693 in the RPL dataset and *R*^2^ = 0.769 in the RPLM dataset) on milk protein synthesis (g/d) during early lactation. Nitrogen metabolism parameters were not affected by feeding RPL or RPLM, either top-dress or when supplemented to deficient diets. Lactation performance did not differ between AA-deficient or AA-adequate diets in response to RPL or RPLM supplementation.

**Conclusion:**

RPL or RPLM showed a positive linear relationship on the lactational performance of dairy cows whereas greater improvement effects were observed during early lactation. Supplementing RPL or RPLM is recommended on deficient-AA diet but not on adequate-AA diet.

## INTRODUCTION

A balance and sufficient supply of amino acids (AA) are driving factors to achieve highly efficient dairy cows′ productivity. In many practical situations, AA deficiency in high-producing dairy cows exists especially the supply of lysine (Lys) and methionine (Met) as the first and second limiting AA [1–3]. Thus, it is a common practice to supplement diets with synthetic Lys and Met. However, Lys and Met are easily degraded by rumen microbes and environment. Therefore, protecting them from rumen microbial and chemical degradation has been chosen as an efficacious strategy to effectively deliver the AA to meet the physiological requirements of lactating dairy cows [4,5]. In the rumen, non-protected AA and true protein are subjected to rumen microbial degradation, deaminated, and are used for rumen microbial protein (MP) synthesis together with carbon skeleton. This highly degradable AA in the rumen is unfavorable to deliver targeted amount of AA into small intestine for absorption and therefore protecting the AA from rumen degradation is critical. Commercially available protected AA products have been developed by industry achieving rumen stability and high bioavailable in the small intestine by coating or encapsulating with pH-sensitive copolymer that are resistant to rumen degradation [6]. The delivery of AA into small intestine as an absorptive site is aimed to increase metabolizable AA supply which is expected to increase milk protein synthesis while reducing N excretion to the environment [7]. Nitrogen (N) source from MP to delivers insufficient AA for optimal protein synthesis in the mammary gland because a certain amount of the AA-N is metabolized into urea-N in the liver. After being released from the liver, urea-N is either excreted via kidney clearance into urine or re-circulated to the gastrointestinal tract. The greater urea-N (plasma urea-N or PUN) in the circulation is an indication of inefficient N use because it has been positively correlated with urinary N excretion [8]. Excretory N, ammonia (NH_3_) volatilization, and nitrous oxide formation are serious concerns for environmental sustainability.

Earlier, feeding post-ruminally available Met via rumen-protected Met (RPM) had been extensively carried out to improve production efficiency in dairy cows following the successful development of commercial RPM products. Progress in this topical study has been made, since first conducted in the 1980s [9]. Driven by the success of RPM development, the available product of rumen-protected Lys (RPL) followed shortly after. However, the production response to dietary RPL has been variable and inconsistent. For instance, improved milk yield due to RPL supplementation was observed in a few studies [7,10], but the latter was only observed during early lactation. Most RPL feeding studies reported a lack of statistically significant results on milk production [11–13]. Additionally, when fed a low crude protein diet (CP) or deficient Lys diets, no consistent results were found in response to RPL supplementation [14–16]. RPM, on the other hand, was demonstrated to linearly increase milk production in a recent meta-analysis [17], although they also provided evidence that RPM did not improve milk production in a discrete analysis. Furthermore, it was suggested that feeding RPLM is more promising to improve production traits and efficiency [18–20], but growing numbers of studies also pointed out little to non-substantial response to RPLM supplementation [21–23].

Lysine and methionine experience multifaceted ruminal microbial degradation and post-ruminal metabolism. Even though they are provided in protected form, interaction with various nutrients and conditions during metabolism makes the prediction of milk protein synthesis challenging. Despite this, it was postulated that there is a linear relationship between duodenal AA supply with plasma AA concentrations including Lys and Met [24], and differential rates of AA delivery into the duodenal sites greatly influence the effect on milk component biosynthesis. So far, knowledge of the accuracy of protected Lys and Met transfer into duodenum, plasma, and milk is often complicated with high variability among studies. The large variability in the substantial body of literature requires a comprehensive examination of possible factors that significantly contribute to the different outcomes. In general, RPL and RPLM supplementation are intended to either increase productivity or maintain productivity as complementary AA-deficient diets. In either case, it is important to assess the relationships of RPL alone or in combination with Met (RPLM) in various conditions, i.e., AA-deficient, or not AA-adequate diets, lactational stages, and supplementary levels on lactation performance. Supplying Lys and Met together as RPLM, theoretically, could provide more balance AA that might have higher effect to improve milk production efficiency. Accordingly, we hypnotized that RPLM supplementation would have greater effect to increase milk production and the efficiency of production and N utilization than RPL in dairy cows. Therefore, the present meta-analysis aimed to examine the relationships of supplemental RPL or RPLM on the plasma AA profile, nitrogen metabolism, and lactational performance of dairy cows.

## MATERIALS AND METHODS

### Literature search

Articles were retrieved from Web of Science ( https://www.webofscience.com/wos/woscc/basic-search), PubMed ( https://www.ncbi.nlm.nih.gov/pmc/), ScienceDirect ( https://www.sciencedirect.com/), and Scopus ( https://www.scopus.com/search/form.uri?display=basic#basic) databases. These databases were selected to assure the quality of articles. A Preferred Reporting Items for Systematic Reviews and Meta-Analyses (PRISMA) protocol was used for bias control during the selection procedure [25]. Keywords “Protected lysine”, “Protected methionine”, and/or "Dairy cows" were applied to all databases. The output titles were imported into an excel spreadsheet for further selection.

### Eligibility and study selection process

**Figure 1:**
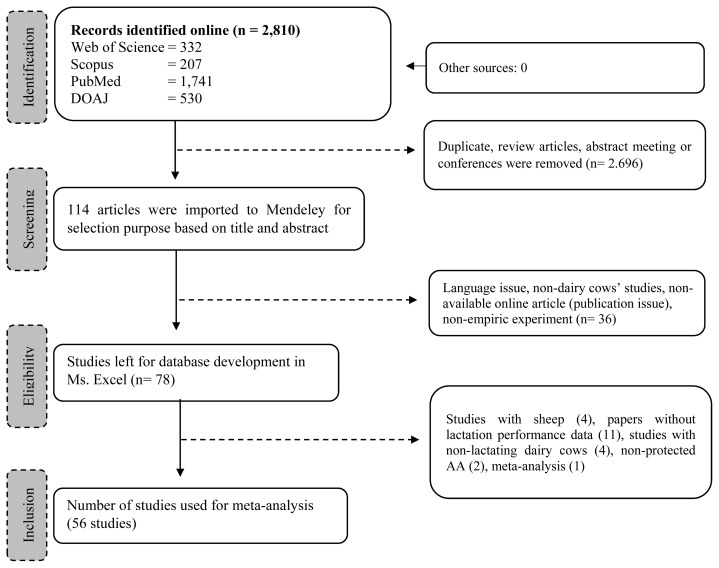
Flowchart of article selection based on Preferred Reporting Items for Systematic Reviews and Meta-Analyses (PRISMA) protocol.

We restricted the inclusion of articles based on the following criteria: i) published as a full-length research article; ii) assessed either dietary RPL and RPLM effects on lactating dairy cows which involved control and treatment group in the experiment; iii) did not include unhealthy animals, toxicology study, or other dietary additives that might interfere with the RPL and RPLM effect; iv) provided sufficient information of how the research was conducted including animal randomization, number of animals, rations and composition, level of inclusion of the dietary treatment, procedure of data collection and analysis, and statistical analysis; v) declared the institutional approval of the use of animals in the study. In addition, only articles published in English were considered in the meta-analysis. No restriction on publication year was applied. The exclusion criteria were as follows: i) no control group; ii) used other dietary interventions that complicated the interpretation of the results; iii) did not measure targeted response variables, especially lactational performance. The selection processes were conducted with six members of the authors. The final selected articles were discussed, and any disagreement was resolved in the discussion. A total of 56 studies comprising 77 experiments were selected and added to a working directory in Mendeley where data extraction was conducted. Details of studies and experimental conditions are available in Table 1 and the representative selection procedure is displayed in Figure 1.

### Data extraction

All relevant information available in the study was extracted into the spreadsheet including first author, year, publishing journal, number of replication, number of animals per replicate, parity, body weight (BW) of the animals, breed, days in milk (DIM) when the experiment was performed, the form of protected AA, company, inclusion levels, and information on the chemical composition of the ration especially CP and net energy for lactation (NE_L_) levels. The responses of interest included lactational performance (milk yield, dry matter intake [DMI], milk/DMI as a measure of feed efficiency), milk components (percentage and yield of milk fat, milk protein, milk lactose, casein, and whey), milk urea nitrogen, somatic cells count, blood profile (glucose, non-esterified fatty acid, blood urea nitrogen), digestibility (dry matter digestibility, organic matter digestibility, nitrogen digestibility, and neutral detergent fiber digestibility), N partitioning, and plasma AA profile. A webplotdigitizer ( https://automeris.io/WebPlotDigitizer/) [68] was employed to generate graphical data. A 3.5% fat-corrected milk (3.5% FCM) and energy-corrected milk (ECM) were calculated from the milk yield and milk component data [69] as follows:


Eq. 1 3.5% FCM=[(0.4324×kg of milk)+(16.216×kg of milk fat)]

Eq. 2 ECM=[(0.327×kg of milk)+(12.951×kg of milk fat)+(7.20×kg of milk protein)]

All the quantitative data were standardized into similar measurement units within the studies. The inclusion levels of RPL and RPLM (g/d) were provided as intestinally available Lys and Met. Some articles have provided such information. For articles that did not provide the intestinally available AA, the calculation was done by multiplying the inclusion levels by the bioavailability specified in the article. This meta-analysis used a cutoff of 5 as the minimum number of studies for each response variable to be included in the analysis.

### Statistical analysis

We performed a meta-regression and meta-analysis to examine the dietary inclusion levels of RPL and RPLM on the lactational performance of dairy cows. All analyses were conducted in SAS 9.4 environment [70] following a similar manner as previously published studies [71,72] with few modifications. Prior to the analyses, outliers were identified by examining the raw data by using PROC REG of SAS. The data points that were deemed identified as outliers, i.e., the value of studentized residuals at −3<t<3 or Cook's distance test −1<t<1 were removed from the dataset.

Meta-regression was conducted using supplementary levels of RPL and RPLM as continuous predictor variables according to Linear Mixed Models by employing PROC MIXED of SAS to assess the effect of their inclusion levels together with other covariates on the response variables of interests. To identify potential covariates, a Spearman correlation matrix was generated using the PROC CORR procedure that contains several candidates of covariates: levels of RPL, RPM, RPLM, levels of CP, NDF, and NE_L_ in the rations, and several targeted response variables: milk yield, FCM, ECM, DMI, milk fat, milk protein, plasma Lys, and plasma Met concentrations. The variables having a Spearman correlation coefficient (|r|) ≥0.3 were considered collinear and thus were not included in the model simultaneously. An arbitrary cut-off value of |r|≥0.4 was set up to identify the influential variables to be included in the model development [3]. In each model, the respective predictors were set as fixed effects while different experiments were stated as a random effect in the random statement of the model [73]. Assessment of the relationship between dietary levels with response variables was investigated by using the following model:


[Eq. 3; full model] $\Delta {ϒ}_{\text{ij}}=\beta_{0}+\beta_{1}{\text{X}}_{\text{ij}}+\beta_{2}{\text{X}}_{\text{ij}}{\;}^{2}+(\beta_{1}\times \beta_{3}\ldots \text{n}){\text{X}}_{\text{ij}}\times {\text{s}}_{\text{i}}+{\text{b}}_{\text{i}}\text{X}+{ɛ}_{\text{ij}},$

[Eq. 4; reduced model] $\Delta {ϒ}_{\text{ij}}=\beta_{0}+\beta_{1}{\text{X}}_{\text{ij}}+\beta_{2}{\text{X}}_{\text{ij}}{\;}^{2}+(\beta_{1}\times \beta_{3}\ldots \text{n}-\text{1}){\text{X}}_{\text{ij}}\times {\text{s}}_{\text{i}}+{\text{b}}_{\text{i}}\text{X}+{ɛ}_{\text{ij}},$

where ∆ϒ _ij_ = estimated outcome of the dependent variable, β_0_ = estimated intercept (fixed effect), β_1_ = coefficient of linear regression of the level of dietary RPL or RPLM (fixed effect), β_2_ = coefficient of the quadratic term of the level of dietary RPAA (fixed effect), X_ij_ = levels of inclusion, the continuous predictor variable, β_3_… β_n_ = covariate from categorical variables, s_i_ = the random effect of the experiment, b_i_ = the random effect of experiment on the regression coefficient of Y on X, and ɛ_i_ = the residual error at ~N(0,σ^2^). The final selected models used an inverse variance matrix as a weighting factor.

A backward stepwise elimination procedure was applied to develop and evaluate the models. In brief, the full model (Eq. 3) was initially assessed involving all covariates that were previously selected using the method described above. In addition, several categorical variables (lactation stage, parity, breed) were also tested in the model, both in a separate (interaction) and parallel function. For each elimination step, one covariate was retrieved, giving the reduced model (Eq. 4) where both full and reduced models were statistically tested using analysis of variance and by comparing the extra sum square F-statistics between the full and reduced models [72]. The covariate was removed at p>0.10. In addition, linear and quadratic terms were tested where the quadratic models were retained when statistically significant, otherwise, the linear model was retained. Selected models with p>0.05 were further evaluated and validated for their performance.

Additionally, a meta-analysis was performed using categorical variables to compare the dietary treatments. The dietary treatments of the experiment involving RPL and RPLM inclusions generally included the control diet with sufficient calculated AA or metabolizable protein (MP) (CON+), control diet deficient in either AA or MP (CON−), and treatment using RPL (RPL group), RPM (RPM group), and RPLM (RPLM group). In the model, they were encoded and stated as fixed effects and the different studies as random effects, following similar manner as described above. The following statistical model was used:


Eq. 5 $${\text{Y}}_{\text{ij}}=\mu +{\text{s}}_{\tau \text{ij}}+\beta \text{a}+(\beta_{\text{a}}\times \beta_{\text{b}}){\text{x}}_{\text{ij}}+\text{s}\beta_{\text{ij}}+{\text{e}}_{\text{ij}}$$

where Y_ij_ = the estimated means of response variable Y, μ = overall mean, S_i_ = random effect of different experiment, β_j_ = fixed effect of treatment group, β_a_×β_b_ = interaction effect between treatment group and covariate, s_τij_ = random interaction between *i* experiment and the *j* treatment group, and e_ij_ = residual error ~N(0, σ^2^). A significant effect was declared at p<0.05 and a tendency was stated when p-value was between 0.05 and 0.10. Tukey-Kramer’s test was used to separate the least square means of the categorical variables. Results of categorical variables were lack of significance on lactational performance and are provided in Supplementary Table S4.

### Model evaluation

The final selected models were evaluated and validated prior to the implementation for analysis using all response variables. Evaluation of the models was aimed to examine the model performance, precision, and accuracy against the observed value obtained from the original studies. Model evaluation was performed following previously published meta-analysis studies [74–76]. For this purpose, milk yield and milk/DMI data were used to test the models. First, mean square prediction error (MSPE) was estimated using the following equation:


Eq. 6 $$MSPE=\sum_{i=1}^{n}\frac{{\left(Y_{obs}-Y_{pred}\right)}^{2}}{n}$$

Where *Y_obs_* and *Y_pred_* are the observed and predicted values of the *i*th observations and n is the sample size (total observations). Using the MSPE, square root of the mean square error (RMSE) was calculated to assess the prediction error of the model as follows:


Eq. 7 $$RMSE=\frac{\sqrt{MSPE}}{\bar{O}}\times 100\%$$

Where *Ō* is the observed mean of the response variable. The RMSE was expressed as a percentage of the observed value of the response variable. In addition, concordance correlation coefficient (CCC) value was used to quantify the accuracy and the precision of the selected models by comparing the deviation between the best-fitted regression line with the reference line (*y* = *x*). The CCC was calculated as follows:


Eq. 8 CCC=r×Cb

Eq. 8a $$Cb=2\times {\left[v+\frac{1}{v}+\mu^{2}\right]}^{-1}$$

Eq. 8b $$\nu ={SD}_{O}/{SD}_{P}$$

Eq. 8c $$\mu =\bar{O}-\bar{P}{\left(SD_{o}\times SD_{p}\right)}^{1/2}$$

Where *r* is Pearson correlation coefficient between observed and predicted values to measure the precision while *Cb* is the correction factor of bias to estimate the accuracy. *SD_O_* and *SD_P_* are standard deviations of observed and predicted values, respectively. The CCC evaluation matrix includes v to measure of scale of fit and μ denotes a location shift measurement. The value of CCC ranges from −1 to +1 where −1 demonstrates a perfect disagreement and +1 indicates a perfect agreement.

## RESULTS

### Description of the selected studies

A total of 56 publications reporting the lactational performance of dairy cows fed supplemental RPL and RPLM were included in the database (Table 1). These publications yield 208 experimental units where 72.9% experiments were conducted on early lactating dairy cows (DIM<100) and the rest were mid to late lactation stage (27.1%; DIM >100). In total, 84.7% of the experiments used Holstein cows, and few used Jersey (7.9%), crossbreed cows, polish red and white, and not reported (7.4%). Smartamine and Ajinomoto rumen-protected AA were the most frequent products identified in the studies, each representing 25.6% and 23.6%, respectively. These commercial products included RPL, RPM, and RPLM. Other commercial products were from Balchem, CJ CheilJedang, Ascor Chimici Srl, Beijing, Evonik, Jefo Inc. Canada, Kemin Industries Inc., Eastman Chemicals Division Research Laboratories, Fiddy feed production technology Cp. Ltd, Beijing, LysiPEARL, Mepron. MetaboLys, H. J. Baker & Bro. Inc., Prince Agri Products, S. Pierce-Sandner, and a few laboratories developed products. RPL treatment was used in most included studies (92.6%) while RPL and RPLM were used in 63.0% of studies.

Descriptive statistics of control and RPL and RPLM treatment are presented in Table 2. Supplementary levels of RPL, RPM, and RPLM ranged from 15.0 to 131.4 g/d, 13.3 to 48.5 g/d, and 20.0 to 139.0 g/d, respectively. Chemical compositions from all studies met the NRC [3] recommendation, according to the CP (16.69±2.29 for control and 16.87%± 2.73% for treatment groups, respectively) and NE_L_ values (1.67±0.09 Mcal/kg dry matter both for control and treatment group) for lactation cows' diet. Overall, data of lactational performance, milk component, digestibility, N partitioning, and plasma essential amino acids (EAA) values are in the expected ranges. The average values N partitioning data were highly variable, but all were still in the acceptable ranges.

### Model selection

A spearman correlation analysis identified collinearity between RPL and RPLM inclusion levels. Thus, we evaluated our meta-regression using those predictors individually for milk yield data as response variables with the largest sample size. As RPM did not show any significant effect on performance data, which was similar to the latest meta-analysis that examined the effect of RPM on lactation performance [17], we decided to exclusively discuss the RPL and RPLM effects and will not further discuss the RPM effect in this meta-analysis. The use of RPL and RPLM against the full dataset resulted in a very small relationship (R^2^<0.10) and lack of significant effects on many of the parameters estimated, because of significant interactions between Early×Mid lactating cows as well as between top-dressed × AA-deficient diets. This provides a rational point to refine the data into specific lactation phase and types of diets (either top-dress or AA deficient) with the consequent reduction in sample size. Analysis of mid-lactation cows resulted in non-significant results on lactational performance data. The covariates analyses showed that CP and NE_L_ had no significant effect on lactational performance for RPL and RPLM sub-datasets (Supplementary Table S1 and S2), thus they were removed from the models. In addition, inclusion of NDF, breed, and parity produced no significant improvement to the model, thus we disregarded those covariates. At this point, a subset of data from experiments that were performed during early lactation and top-dress setting was kept for further model evaluations.

**Figure 2:**
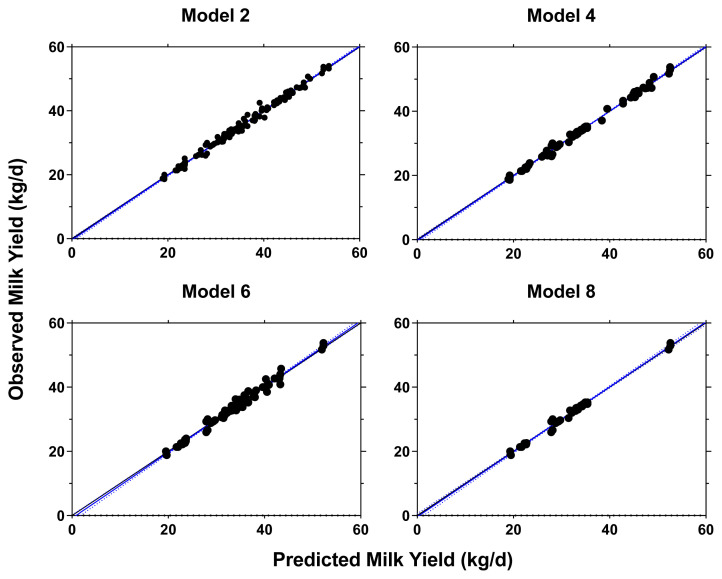
Observed vs predicted plots for milk yield (kg/d) prediction equation using different models. Model 1 = RPLM (full); Model 2 = RPLM (top-dressed); Model 3 = RPLM + CP (top-dressed); Model 4 = RPL (early lactation); Model 5 = RPLM (early lactation); Model 6 = RPLM + CP (early lactation). The blue (95% confidence interval) and black solid lines represent the fitted regression line for the relationship between the predicted and observed values and the identity line (y = x), respectively. RPLM, rumen-protected lysine + methionine; RPL, rumen-protected lysine; CP, crude protein.

Using the RPL and RPLM datasets, 8 retained models were evaluated and the result is provided in Supplementary Table S3. Four models were chosen by considering the model performance from 8 regression models where the result of model performance evaluation is presented Table 3. The selected models were all in good fit with low RMSE (2.03% to 2.71%), high CCC (>0.99), and high precision outcome as shown from the regression plots between predicted vs observed values (Figure 2). The models also indicated that RPL and RPLM had equal statistical power on the lactational performance data.

### Meta-regression

As shown in Table 4, the improvement of estimated parameters of interest was observed as indicated from the RMSE and *R*^2^, after model selection and evaluation using a subset of data. Milk yield, 3.5% FCM, and ECM were linearly increased in response to the increased levels of RPL (p<0.001) with the highest correlation coefficient on milk yield (*R*^2^ = 0.123). Increasing RPL supply did not affect DMI but had a positive linear relationship with milk yield/DMI (p = 0.013; *R*^2^ = 0.031) and ECM/DMI (p<0.001; *R*^2^ = 0.048) as a measure of dairy efficiency although the magnitude effects were weak. No milk fat, milk protein, and milk lactose percentages were affected by the doses of RPL supplementation (p>0.05). However, increasing the supply of available RPL on absorptive sites linearly increased the yield of milk fat (p<0.001; *R*^2^ = 0.025), milk protein (p<0.001; *R*^2^ = 0.214), and milk lactose (p<0.001; *R*^2^ = 0.114). Among all EAA’ concentrations measured in the plasma, Lys concentration was the only AA affected by dietary RPL (p = 0.021; *R*^2^ = 0.067).

In general, RPLM had a relatively similar response effect with RPL on the variables included in the analysis with few differences. RPLM exhibited a linear increase (p = 0.028; *R*^2^ = 0.195) in milk yield but had no effect on FCM and ECM (g/d) when supplemented during early lactation (Table 5). Similar to RPL, increasing RPLM supply positively correlated with the increased in dairy efficiency as shown in the milk yield/DMI (p = 0.019; *R*^2^ = 0.229) and ECM/DMI (p = 0.036; *R*^2^ = 0.242) and had no effect on the percentage of milk fat, milk protein, and milk lactose composition (p>0.05). Levels of RPLM had linear relationship with milk protein synthesis (p<0.001; *R*^2^ = 0.479) but had no relationship with milk fat yield and milk lactose yield. Increasing RPLM levels did not affect overall AA’ concentration in the plasma except for Met concentration that positively correlated (p = 0.004; *R*^2^ = 0.100). Interestingly, plasma Lys showed strong linear relationship with milk protein synthesis (p<0.001; *R*^2^ = 0.769), similar with the data of RPL.

**Figure 3:**
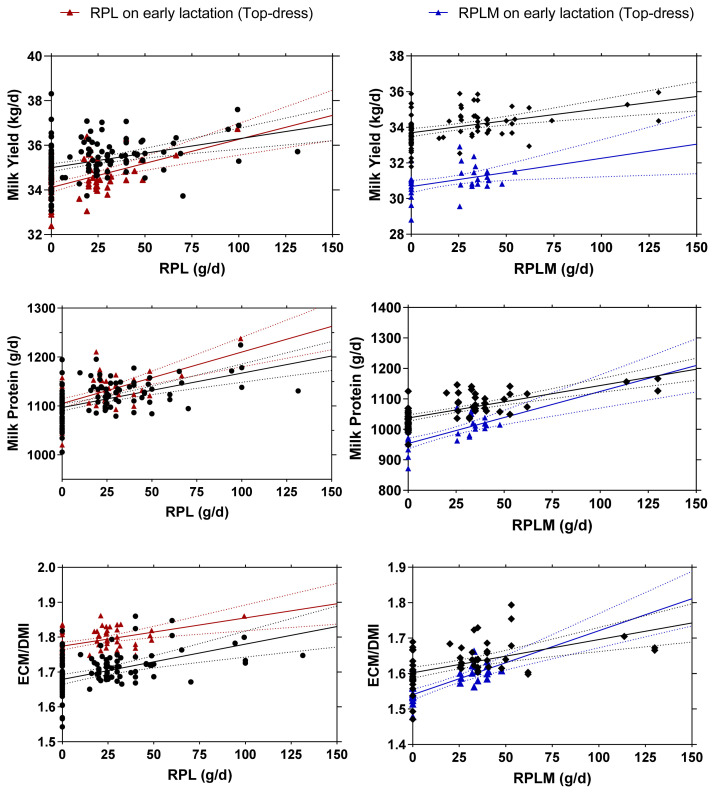
Relationship between plasma RPL (A) or RPLM (B) inclusion levels on milk yield (kg/d), milk protein (g/d), and ECM/DMI in early lactating dairy cows. A = regression plots comparison between full dataset (black solid line; equation for milk yield: y = 35+0.013x; p<0.001; R^2^ = 0.139, milk protein: y = 1,097+0.702x; p<0.001; R^2^ = 0.248, ECM/DMI: y = 1.67+0.001x; p<0.001; R^2^ = 0.152) vs top dress only dataset (blue solid line; equation for milk yield: y = 34.1+0.021x; p<0.001; R^2^ = 0.279, milk protein: y = 1,105+1.051x; p<0.001; R^2^ = 0.366, ECM/DMI: y = 1.77+0.0008x; p<0.001; R^2^ = 0.200) from RPL regression model. B = regression plots comparison between full dataset (black solid line; equation for milk yield: y = 33.7+0.014x; p<0.001; R^2^ = 0.195, milk protein: y = 1,037+1.067x; p<0.001; R^2^ = 0.479, ECM/DMI: y = 1.60+0.001x; p<0.001; R^2^ = 0.242) vs top dress only dataset (blue solid line; equation for milk yield: y = 30.7+0.016x; p<0.001; R^2^ = 0.158, milk protein: y = 954+1.709x; p<0.001; R^2^ = 0.511, ECM/DMI: y = 1.54+0.002x; p<0.001; R^2^ = 0.599) from RPLM regression model. The regression analysis was performed according to mixed regression models. RPL, rumen-protected lysine; RPLM, rumen-protected lysine + methionine; ECM, energy-corrected milk; DMI, dry matter intake.

**Figure 4:**
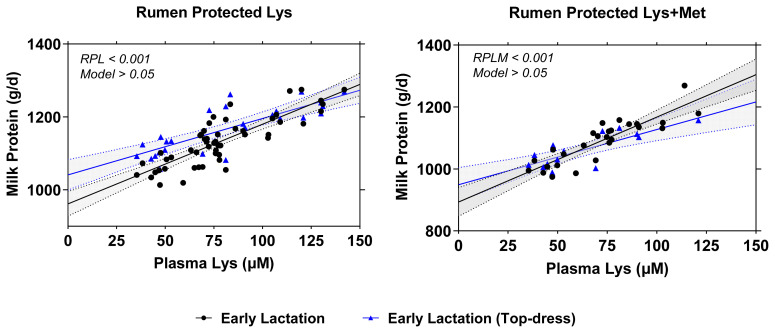
Relationship between plasma Lys concentration and milk protein synthesis (g/d) using RPL (A) and RPLM (B) datasets in early lactating dairy cows. A = regression plots comparison between full dataset (black solid line; y = 961.4+2.185x; p<0.001; R^2^ = 0.693) vs top dress only dataset (blue solid line; equation: y = 1,041+1.55x; p<0.001; R^2^ = 0.657) from RPL regression model. B = regression plots comparison between full dataset (black solid line; y = 893.3+2.741x; p<0.001; R^2^ = 0.769) vs top dress only dataset (blue solid line; equation: y = 949.1+1.781x; p<0.001; R^2^ = 0.653) from RPLM regression model. The regression analysis was performed according to mixed regression models. RPL, rumen-protected lysine; RPLM, rumen-protected lysine + methionine.

Figure 3 illustrates the comparison results between two different models in either RPL or RPLM dataset. Overall, RPL and RPLM supplementation during early lactation, either using full dataset or top-dress design only showed a similar empirical power to increase milk yield, milk protein synthesis (g/d), and ECM/DMI (p<0.001). The similar behaviors were also observed on the relationship between plasma Lys concentrations and milk protein synthesis in all models with no significant differences (p>0.05) among all examined models (Figure 4).

In the present meta-analysis, the effects of RPL or RPLM on all response variables were not observed (p>0.05) by considering the treatment group as categorical data (Supplementary Table S4). No interaction effects were also found for Group× Diet (p>0.05).

## DISCUSSION

Increasing Lys and Met availability in a balanced manner for dairy cows is expected to increase efficiency of milk protein synthesis or maintain productive performance. It is because Lys and Met are the first two limiting AA in dairy cows [77] in which deficient supply or imbalance between those two would unfavorably change AA metabolism and energy partitioning which eventually disrupts milk synthesis. Supplying Lys and Met in the form of RPL or RPLM is therefore essential to meet the AA requirement for optimum milk protein synthesis in the mammary gland and improves milk production [62]. Nevertheless, evidence from the first meta-analysis examining supplementation of post-ruminally available Lys or Met showed that the response to both increasing Met and Lys intakes was a declining efficiency of milk protein synthesis [1]. As studies investigating the production response to RPL and RPLM evolved, comprehensive examination of their effects on production traits and milk component synthesis is required. Our findings revealed that the status of AA or MP deficiency and lactation phase greatly influenced the prediction models. Under deficiency of Lys or Met, or when dietary CP or NE_L_ were reduced, RPL supplementation was most likely to maintain production performance while fewer studies reported decreasing in milk production and milk component [21,38].

In the present meta-analysis, higher supply of intestinally available RPL in early lactating cows resulted in linear increase in milk production (kg/d), FCM, ECM, and overall dairy efficiency as measured by milk yield/DMI and ECM/DMI. Linear increases were also observed for the yield of milk protein, milk fat, and milk lactose production (g/d). Estimation using the result of regression equation indicated that an increase of 1.30 kg/d milk yield and 53.5 g/d milk protein was obtained by supplementing 100 g/d of intestinally available RPL when Lys supply from basal diet held constant or considered as adequate, assuming the absorption rate or bioavailability to be at least as guaranteed by producers (~50%). Experiments that directly measure the bioavailability of Lys are scarce and considerable variability was reported, such as [77] who reported the bioavailability of Lys ranged between 11% to 67%, in which one of three products had lower bioavailability than the company recommendation. Discrepancies of Lys bioavailability were also reported by different studies [78,79] even for products with similar protection methods, which might, in part, explain the effect of RPL on milk production and component synthesis. The variability of RPL and RPLM bioavailability might also be attributed to the protection methods. There are several methods that have been developed by manufacturers to protect Lys and Met from rumen microbes and environment such as coating the Lys and Met using ethyl-cellulose, pH-sensitive co-polymer, calcium soaps, hydrogenated fatty acids, and triglycerides [78]. The RPL and RPM escapes degradation in the rumen because the coating method especially using pH-sensitive co-polymer is effective preventing rumen bacterial attack and it is highly resistant to the rumen pH and rumen environment [78] and therefore increases their availability to meet the animal requirement. Different co-polymers could possibly affect the bioavailability.

The increased milk production was in line with the finding of Fehlberg et al [62] who reported a higher milk increase than that of the estimated value (+3 kg/d) by delivering 99.3 g/d of absorbable RPL for early lactating dairy cows. Similarly, Robinson et al [52] also reported +2 kg/d increase in milk production of dairy cows during early lactation. The lower estimated result from our meta-analysis was expected due to the variability within studies. Such variability could also be due to the different length of RPL supplementation. Several studies, for instance, demonstrated that 90 d and 280 d RPL supplementation increased lactational performance [32,33] while shorter supplementation period produced inconsistent findings [11,23,55]. Our findings revealed that lactation stage influenced the outcome variables. Supplementation of RPL in early but not in mid or late lactating dairy cows is likely to increase milk production plausibly because the cows in the early postpartum are most likely deficient in nutrient supply (negative energy balance) including MP and AA. Therefore, providing intestinally available Lys at this period would help meeting the needs of Lys for milk production as evident from several studies [10,62]. In mid-lactating cows, on the other hand, protected Lys and other AAs supplementation resulted in none to small effect on production traits as the cows have sufficient AA supply from the diet [21,23,80]. In this situation, it is not surprising if supplementary Lys or Met increased plasma Lys [23] but not milk protein yield because the plasma Lys is re-routed into body protein flux. Studies have observed a decrease of Lys and Met efficiencies for milk protein synthesis especially when the supplemental doses exceed the requirement, as previously suggested [1,5]. This was supported by the decrease of the utilization of Lys (from 39% to 25%) and Met (44% to 12%) for milk protein synthesis in response to increasing Lys and Met supply [1] and an overall lower N utilization efficiency when dietary CP increases [81]. There are several factors that could explain the efficiency of Lys and Met supply to lactating dairy cows: energy and MP levels or status, AA balance, DMI, protecting efficiency, and supplementation methods. After absorption, metabolizable AA undergoes complete oxidation for energy production and the rest would be utilized for milk production [5]. In addition, incomplete endogenous AA reabsorption and oxidation of AA in the epithelial cells also contribute to the AA losses that lead to low efficiency of AA utilization [1,82]. Under energy deficiency status, cows would utilize the AA for energy production via gluconeogenesis and AA breakdown would increase in the condition of excessive AA supply resulting in higher urinary N and lower N use efficiency [83]. Imbalance of AA supply could also prevent the absorption of other AA, as suggested by Baumrecker [84].

A higher increase of ECM during early lactation as a result of dietary RPL is validated in our meta-analysis, indicated by the higher slope of ECM than that of milk yield from the regression equation. This was mainly explained by the pertinent increase in milk fat yield in response to dietary RPL. It was suggested that milk fat synthesis increased when Lys or Met are oversupplied [34] via diverting the excessive AA into *de novo* milk fat synthesis. Increasing milk fatty acids in the study conducted by Robinson et al [52] and by Fehlberg et al [62], likely resulted from greater de novo milk FA production as suggested by Woolpert et al [85] exacerbate this hypothesis. This was similar to our result where elevating RPL levels linearly increased milk fat yield at the rate of 0.928 g/d as per 1 g/d Lys increase. However, it should be noted that an enhancement in milk production and synthesis of protein and fat would be observed only when MP supply met physiological requirements of dairy cows, as evidenced by the contrasting results found by Lee et al [11] and Fehlberg et al [6] in which they had different MP supply from their formulated diet (800 vs 1,190 g/d). Additionally, the increase in milk lactose yield could be explained by gluconeogenesis of AA into lactose similar to the fat when Lys or Met supply excess of the minimum requirement.

Regarding RPLM, we found similar results with RPL where increasing RPLM level produced a linear increased on milk yield and milk protein synthesis (Table 5). In many studies, lack of effect on milk production was reported with RPM alone [17,59], probably because Met was not the first limiting AA. Therefore, the production response was more sensitive to RPL supplementation as Lys is known to be the first limiting AA. Many other plausible reasons might be related to the basal MP or Met level, diet composition, and adequacy status [2,43,52]. This might also explain why the improvement effect was little to none in RPLM compared to RPL. However, it might be taken into consideration that it does not necessarily compare their effects due to the scarcity of studies that directly compared RPL and RPLM. In most studies included in our meta-analysis, RPL and RPLM were obtained from independent studies except in a few in very few experiments. Additionally, RPLM levels failed to increase milk fat and milk lactose but did increase milk protein yield. Earlier studies reported higher milk protein production from cows in two different locations supplemented with RPLM [10,86] with the condition of adequate CP in the basal diets for microbial growth. A greater nutrient supply especially MP and AA exceeding the requirement for milk protein synthesis would be circulated for body protein at a different priority. This is especially true in the case of postpartum and early lactating cows where increasing protein or AA supply would reduce alveolar cell apoptosis and is important to maintain mammary cell functions for milk synthesis [87]. It is probably also relatable to question whether the efficiency of RPL or RPLM depends on the protection methods. However, our meta-analysis did not compare the protection methods as they were not very heterogeneous; more than 90% of the studies described similar protection methods regardless of the differences among producers, despite discrepancies existing for this. Fagundes et al [66] demonstrated that three different forms of commercially protected Lys at recommended doses did not affect milk performance, component, and efficiency of mid-to-late lactating dairy cows with a potential increase in N use efficiency [66]. They also found a lack of increasing AA delivery to plasma EAA concentrations, although the supplementary levels of MP were higher with relatively balanced Lys for treatment group.

Furthermore, the linear increase in plasma Lys concentrations which concurrently increased milk protein yield in linear manner support previous hypothesis that level of deliverable Lys in the intestinal absorptive sites would be metabolized in the plasma and mammary gland for milk protein and milk lactose synthesis [10]. A greater relationship between plasma Lys and milk protein suggests that the effect was almost entirely postabsorptive, similar when glucose was supplemented in the duodenal site. Nevertheless, the overestimation dietary absorbable Lys or Met might result in an underestimation of milk production and component synthesis. For instance, Robinson et al [10] found 49% to 63% lower intestinal deliverable Lys from the calculated target. They reported different responses of early vs mid-lactating cows whereas the RPL increased the production of milk, fat, and true protein in the early lactation but similar production traits during mid-lactation. Also, a meta-analysis estimated that constant increase in either Lys or Met is likely to decrease the efficiency of AA conversion into milk protein [1]. Furthermore, Lys is categorized as Group II AA where the conversion of Lys into per unit of milk protein requires more the requirement which is plausible to explain the underestimation of milk protein as a function of plasma Lys concentrations although the relationship was considered as strong (R^2^ = 0.708). For the conversion of Lys as measured by U:O, however, discrepancies exist in the literature from 1.27 [82] to 1.38 and 1.42 [88] depending on CP levels of the diets and the physiological state of the cows.

As a major net user of EAA supply, the mammary gland plays a greater role in EAA partitioning than other organs even though overall routes of EAA from intestine, bloodstream, liver, and mammary gland itself are not straightforward [82]. Lys and Met are converted into milk protein components at different rates according to the individual AA uptake-to-output ratio in the milk protein. After intestinal absorption, Lys is metabolized rapidly into the mammary gland for protein synthesis especially under Lys-deficient diet. Therefore, it is not surprising that in many studies plasma Lys concentration did not change with RPL supplementation [50,53,57]. Protein synthesis in the mammary gland is also dependent on dietary non-starch carbohydrate or energy and the availability of glucose for absorption. It is well known that energy sources determine the yield of MP in the rumen which contributes to approximately 50% of intestinal protein flux [89]. In addition, the availability of absorbable glucose in the intestine was suggested as a driving factor of ribosomal protein S6 (rpS6) activation in the mammary gland and influence metabolic process of milk protein synthesis [90]. Therefore, in a study designed to increase AA delivery into the intestine, it is critical to consider the proportion of AA to starch or energy supply to predict the mechanistic effect more accurately.

Despite this study suggesting a linear relationship for most of the parameters in response to increasing dietary RPL, it should be understood that the applicability of the equation is restricted to the maximum RPLM or RPL used in the dataset. In continuous incremental supply, however, the logistic model and or segmented regression would explain better predictability when the maximum AA supply beyond and under the breakpoint would result in constant efficiency [91]. In our modeling, linear models had better performance than quadratic, logistic, and segmented regression models.

In the present meta-analysis, the effects of supplementary levels of RPL and RPLM on N metabolism were not found by using full dataset. The insignificant effect was mainly due to the high cross-studies variance. In addition, it was also driven by lower N intake especially when RPL or RPLM substituted to MP-deficient diet, as reported in several studies [53,58,66]. Although it was suggested that supplementation of RPL or RPLM on non-deficient diets could improve N use efficiency, it is a limitation of the present meta-analysis having relatively small sample size for N metabolism data. Future modelling studies to assess the relationship between rumen protected AA supplementation and N use efficiency would help directing how to improve the efficiency and lower N excretion.

## CONCLUSION

In conclusion, our meta-analysis demonstrates that increasing either intestinally available RPL or RPLM during early lactation of dairy cows increased lactational performance as shown by the linear increase of milk yield, feed efficiency (ECM/DMI), and milk protein synthesis in which RPLM had a better prediction model than RPL. The linear increase observed on the lactation performance by RPL and RPLM was not different between adequate and AA-deficient diets. In addition, higher efficiency of post-absorption effects of RPL and RPLM on milk protein synthesis as shown by a strong linear relationship between plasma Lys and milk protein synthesis. Therefore, it is recommended to supplement RPL or RPLM to help improve lactational performance of dairy cows when Lys or Met is deficient in the diet, but it is not recommended in adequate AA diets.

## Figures and Tables

**Table 1:** Studies included in the meta-analysis

No	Study	Period (d)	Breed	Parity	DIM	Products	Bioavailability (%)^1)^	Inclusion level (g/d)^2)^		

								RPLM	RPL	RPM
1	Donkin et al [26]	28	Holstein	Multiparous	98–158	Smartamine	80	55	40	15
2	Chow et al [27]	21	Holstein	Mixed	36	S. Pierce-Sandner	n/a	26.38	19.99	6.39
3	Canale et al [28]	21	Holstein	Mixed	50	Smartamine	80	35	20	15
4	Guillaume et al [29]	128	Holstein	Mixed	22	Eastman Chemicals Division Research Laboratories	n/a	54.52	39.48	15.04
5	Karunanandaa et al [30]	21	Jersey	Mixed	50–60	n/a	n/a	32	24	8
6	Colin-Schoellen et al [31]	84	n/a	Multiparous	40	Smartamine	80	40	30	10
7	Robinson et al [32]	280	Holstein	Multiparous	15–28	Ajinomoto	64	25.5	19	6.5
8	Han et al [33]	106	Holstein	Multiparous	92	Ajinomoto	64	66.6	66.6	0
9	Piepenbrink et al [34]	14	Holstein	Multiparous	128	Ajinomoto	64	139	106	33
10	Blauwiekel et al [35]	21	Holstein	n/a	97	Ajinomoto	64	15	15	0
11	Bremmer et al [36]	28	Jersey	Mixed	92	Smartamine	80	16.2	6	10.2
12	Xu et al [37]	301	Holstein	Multiparous	−21	Ajinomoto	64	53	40	13
13	Dinn et al [38]	28	Holstein	n/a	60	Smartamine	80	14.65	7.5	7.15
14	Robinson et al [39]	140	Holstein	Multiparous	15–28	Ajinomoto	64	113.7	65.2	48.5
15	Nichols et al [40]	28	Holstein	Multiparous	57	Smartamine	80	26	20	6
16	Bertrand et al [41]	126	Jersey	Mixed	7	Smartamine	80	20	10	10
17	Pisulewski and Kowalski [42]	14	Polish Red and White	multiparous	56	Smartamine	80	47.75	24.5	23.25
18	Robinson et al [43]	28	Holstein	multiparous	50	Ajinomoto	64	32.8	24.6	8.2
19	Bateman et al [44]	60	Holstein	multiparous	55	Prince Agri Products	25.8	35	25	10
20	Liu et al [45]	28	Holstein	multiparous	83	Smartamine	80	65	50	15
21	Misciattelli et al [46]	84	Holstein	Multiparous	73	Smartamine	80	26	0	26
22	Erasmus et al [47]	98	Holstein	Multiparous	22–121	Smartamine	80	73.968	69	4.968
23	Socha et al [48]	119	Holstein	Multiparous	−14	Smartamine	80	26.2	16	10.2
24	Watanabe et al [49]	119	Holstein	multiparous	126	Ajinomoto	64	22.5	16	6.5
25	Třináctý et al [18]	14	Holstein	multiparous	n/a	n/a	n/a	29.9	11.7	18.2
26	Swanepoel et al [50]	28	Holstein	multiparous	262	Ajinomoto	64	40	40	0
27	Wang et al [51]	56	Chinese Holstein	Mixed	120	Archer Daniels Midland Company	88	105	105	0
28	Robinson et al [52]	28	Holstein	Mixed	48	Ajinomoto	64	0	24	0
29	Robinson et al [10]	56	Holstein	Mixed	48	Ajinomoto	64	94.4	94.4	0
30	Appuhamy et al [11]	7	Holstein	Multiparous	54	Ajinomoto	64	33	21	12
31	Lee et al [53]	70	Holstein	Mixed	95	AminoShure-L	64	130	100	30
32	Li et al [14]	40	Chinese Holstein	multiparous	76	n/a	35	60	40	20
33	Paz et al [54]	21	Holstein	Multiparous	62	AminoShure-L	64	60	60	0
34	Bernard et al [55]	21	Holstein	Mixed	111	Evonil Industries	80	37	37	0
35	Vargas-Rodriguez et al [56]	35	Holstein	Mixed	38	LysiPEARL	50	64.1	48.8	15.3
36	Arriola Apelo et al [57]	15	Holstein	Mixed	189	AminoShure-L	64	56.707	42.063	14.644
37	Pereira et al [15]	21	Holstein	multiparous	334	AminoShure-L	64	89.87	52.25	37.62
38	Lee et al [58]	28	Holstein	multiparous	102	Mepron	72	124	100	24
39	Amrutkar et al [20]	120	Crossbred cows Bos taurus x Bos indicus	Multiparous	n/a	LysiPEARL	50	25	20	5
40	Awawdeh [59]	56	Holstein	multiparous	154	n/a	20.5–25.5	30	0	30
41	Giallongo et al [21]	49	Holstein	Mixed	30	Ajinomoto	64	70.2	70.2	0
42	Liu et al [60]	40	Chinese Holstein	Mixed	75	Fiddy feed production technology Cp. Ltd, Beijing	35	32	32	0
43	Pereira et al [22]	21	Holstein	Mixed	97	Ajinomoto	64	50	37.5	12.5
44	Bailey et al [12]	18	Holstein	multiparous	94	RPL prototype	50	31.5	31.5	0
45	Lee et al [11]	22	Holstein	Mixed	2	USA Lysine	90	37	26	11
46	Weiss [61]	28	Holstein	Mixed	91–135	MetaboLys, H. J. Baker & Bro. Inc., Westport, CT	70–80	84	70	14
47	Girma et al [13]	21	Holstein	multiparous	−21	Ascor Chimici Srl, Beijing	44	40	40	0
48	Fehlberg et al [62]	56	Holstein	multiparous	−28	Ajinomoto	64	99.36	99.36	0
49	Morris and Kononoff [63]	28	Jersey	multiparous	91	Ajinomoto	64	31	31	0
50	Mavrommatis et al [64]	75	pure Chios breed	n/a	50	LysiGEM	63	5	5	0
51	Lobos et al [7]	28	Holstein	multiparous	87	Ajinomoto	64	48	48	0
52	McLain et al [16]	28	Jersey	multiparous	226	Ajinomoto	64	26.65	26.65	0
53	Zang et al [23]	21	Holstein	multiparous	138	Ajinomoto	64	31	15	16
54	Malacco et al [65]	28	Holstein	Mixed	192	n/a	24.4	20	20	0
55	Fagundes et al [66]	70	Holstein	Multiparous	151	Ajinomoto	64	50	50	0
56	Wang et al [67]	56	Holstein	Multiparous	124	KDQ Technology Co. Ltd	80	84.6	66	18.6

DIM, days in milk; n/a, information is not available.

1) Bioavailability (%) is defined as intestinally available and is provided based on the guaranteed information from the company or according to the reported values from the original article. Four studies did not provide bioavailability information (n/a) but they specified the deliverable or intestinally available amount (g/d) of the rumen protected lysine and methionine.

2) Provided as estimated intestinally available (g/d).

**Table 2:** Descriptive statistics

Parameters	Control					Protected-Lys and Met				
	
	N	Mean	SD	Min	Max	N	Mean	SD	Min	Max
Period (d)	94	60.10	64.72	7.00	301.00	127	64.50	69.59	7.00	301.0
BW (kg)	57	583.1	145.8	58.10	733.00	82	574.1	160.3	48.00	750.0
Treatment (g/d)										
RP Lys+Met	87	0.00	0.00	0.00	0.00	116	41.03	29.99	20.0	139.0
RP Lys	87	0.00	0.00	0.00	0.00	116	32.29	27.52	15.0	131.4
RP Met	87	0.00	0.00	0.00	0.00	116	8.74	9.41	13.3	48.5
Chemical composition of diets										
DM (%)	67	58.53	12.00	37.40	90.39	88	59.09	13.63	37.40	90.40
OM (%)	14	83.65	19.79	37.30	94.10	21	84.12	19.71	37.30	94.10
CP (%)	86	16.69	2.29	10.18	30.80	114	16.87	2.73	10.18	31.40
NDF (%)	73	32.76	4.02	25.40	41.70	94	32.73	4.55	21.88	41.70
ADF (%)	71	20.32	3.38	13.80	32.40	87	20.60	3.86	13.80	32.40
NE_L_ (Mcal/kg DM)	54	1.67	0.09	1.47	1.87	63	1.67	0.09	1.47	1.87
Production traits										
Milk yield (kg/d)	90	32.29	10.32	1.52	53.20	120	32.85	10.53	1.37	54.00
4% FCM (kg/d)	86	33.71	8.96	3.19	53.70	116	34.05	9.25	2.81	52.90
ECM (kg/d)	80	34.17	9.71	3.00	54.50	107	34.40	10.40	2.65	53.20
DMI (kg/d)	88	21.43	5.54	2.18	29.80	118	20.96	6.18	2.20	30.10
Milk yield/DMI	87	1.50	0.38	0.60	2.67	114	1.53	0.36	0.53	2.55
FCM/DMI	41	1.55	0.41	1.00	2.83	49	1.57	0.39	1.02	2.86
ECM/DMI	37	1.67	0.46	1.11	2.89	51	1.66	0.40	1.07	2.86
Milk fat (%)	86	3.92	0.89	2.57	10.00	115	3.90	1.05	2.46	10.60
Milk protein (%)	84	3.19	0.35	2.67	4.30	113	3.20	0.31	2.62	4.30
Milk lactose (%)	60	4.82	0.25	4.31	6.40	84	4.81	0.28	4.18	6.40
Milk fat (g/d)	82	1269	367	111	2650	109	1273	368	137	2800
Milk protein (g/d)	80	1035	261	67	1826	107	1056	277	59	1876
Milk lactose (g/d)	55	1655	480	99	2530	75	1644	516	87	2570
Casein (%)	24	3.40	0.93	1.95	4.90	32	3.53	0.91	2.06	5.10
Whey (%)	10	0.11	0.05	0.08	0.25	13	0.11	0.04	0.07	0.25
SCC (10^3^)	25	231.40	118.39	67.80	480.0	33	218.09	111.73	38.90	485.0
MUN (mg/dL)	40	12.73	2.77	6.90	18.57	52	12.51	3.00	6.39	18.60
Digestibility (%)										
DM	18	67.87	6.32	58.20	81.70	22	67.58	6.52	59.30	82.60
OM	14	66.89	3.92	59.00	72.30	15	67.09	4.18	60.10	72.20
N	20	62.14	8.40	34.93	76.40	24	60.84	10.82	34.50	78.60
NDF	16	44.28	9.35	29.00	62.40	18	45.81	9.21	29.70	63.50
ADF	12	42.04	12.92	21.80	63.40	13	44.46	13.11	23.20	62.20
N Partitioning (g/d)										
N intake	18	545.41	200.04	50.00	824.00	22	546.08	180.19	59.20	836.00
Urinary N	14	204.29	68.46	92.00	323.00	18	193.72	65.56	87.00	325.00
Fecal N	12	217.17	45.72	158.00	287.00	16	209.06	49.28	151.00	289.00
Milk N	14	166.39	33.80	124.00	248.00	19	166.29	31.44	119.00	245.00
N Balance	11	51.95	27.86	15.00	95.00	13	45.14	27.88	14.00	106.00
Essential AA (μM)										
Arg	44	83.56	30.21	44.90	182.00	56	83.06	28.11	42.80	189.00
His	42	47.59	25.68	16.10	176.80	52	45.90	22.93	19.20	171.10
Ile	44	102.82	29.07	30.80	157.00	56	101.93	27.18	32.90	147.12
Leu	40	150.89	55.17	78.90	367.60	52	146.60	57.37	87.00	408.20
Lys	44	72.94	20.03	35.40	131.00	56	81.00	23.94	38.30	142.00
Met	44	24.31	19.33	7.00	137.33	56	26.74	10.04	9.10	50.70
Phe	44	47.15	12.54	19.70	78.10	56	46.16	12.87	20.60	79.30
Thr	40	88.75	29.29	27.60	179.70	47	109.39	133.30	25.50	981.00
Val	43	210.56	63.33	77.80	321.00	55	210.25	59.20	79.80	350.00

N, sample size; SD, standard of deviation; OM, organic matter; CP, crude protein; NDF, neutral detergent fiber; ADF, acid detergent fiber; DMI, dry matter intake; ADG, average daily gain; CH4, methane production; FCM, fat-corrected milk; NEFA, non-esterified fatty acid; BHBA, beta-hydroxybutyric acid; BUN, blood urea nitrogen; SCC, somatic cells count; Arg, arginine; His, histidine; Ile, isoleucine; Leu, leucine; Lys, lysine; Met, methionine; Phe, phenylalanine; Thr, threonine; Val, valine.

**Table 3:** Model performance of selected models

Model statistics	Selected models^1)^			

	Model 2	Model 4	Model 6	Model 8
β_0_	34.97	34.31	33.55	30.66
SE (β_0_)	1.186	1.781	1.192	1.968
β_1_	0.015	0.011	0.011	0.011
SE (β_1_)	0.003	0.006	0.005	0.008
p-value	<0.001	0.022	0.046	0.154
Model performance				
*v*	1.030	1.041	1.057	1.016
*μ*	−0.002	0.000	0.000	0.001
*Cb*	1.000	0.999	0.998	1.000
*r*	0.996	0.997	0.992	0.996
CCC	0.995	0.997	0.990	0.996
MSPE	0.672	0.489	0.843	0.495
RMSE	2.355	2.034	2.713	2.237
AIC	698	366	446	194
R^2^	0.134	0.276	0.001	0.089

RPL, rumen-protected lysine; RPLM, rumen-protected lysine methionine; β_0_, overall intercept; SE (β_0_), standard errors of intercept; β_1_, slope for either RPL or RPLM; SE (β_1_), standard error of the slope; *Cb*, model accuracy; *r*, Pearson correlation coefficient; CCC, concordance correlation coefficient; MSPE, mean square prediction errors; RMSE, root-means square error; AIC, Akaike information of criterion; R^2^ = regression coefficient.

1) Model 2, RPL (early lactation data); Model 4, RPL (early lactation data and top-dress); Model 6, RPLM (early lactation data); Model 8, RPL (RPLM (early lactation data and top dress).

**Table 4:** Results of meta-regression analysis of the effects of levels of RPL supplemented during early lactation of dairy cows on production performance and plasma amino acids concentrations

Response variable	N	Parameter estimates				Model statistics			
	
		β_0_	SE (β_0_)	β_1_	SE (β_1_)	p-value	AIC	RMSE	R^2^
Production performance									
Milk yield (kg/d)	144	35.04	1.159	0.013	0.003	<0.001	743	0.92	0.123
3.5% FCM (kg/d)	142	35.20	1.214	0.021	0.004	<0.001	805	1.76	0.060
ECM (kg/d)	131	36.33	1.246	0.021	0.004	<0.001	726	1.60	0.094
DMI (kg/d)	140	22.10	0.53	−0.0001	0.002	0.973	549	0.51	0.000
Milk yield/DMI	140	1.62	0.05	0.0004	0.0002	0.013	−81.7	0.05	0.031
ECM/DMI	140	1.67	0.06	0.0008	0.0002	<0.001	−57	0.06	0.048
Milk fat (%)	141	3.78	0.07	0.001	0.001	0.068	154	0.19	0.000
Milk protein (%)	137	3.17	0.043	0.0001	0.00032	0.505	−78	0.07	0.006
Milk lactose (%)	93	4.77	0.023	0.0001	0.0001	0.440	−187	0.03	0.000
Milk fat (g/d)	135	1,308	44.2	0.928	0.266	0.001	1,821	111.7	0.025
Milk protein (g/d)	131	1,102	39.19	0.535	0.091	<0.001	1,575	35.48	0.214
Milk lactose (g/d)	89	1,773	81.79	0.736	0.157	<0.001	1,152	51.64	0.114
MUN (mg/dL)	55	13.30	0.558	−0.001	0.005	0.874	222	0.76	0.002
Plasma EAA									
Arg	57	88.18	9.017	0.021	0.027	0.446	457	5.00	0.000
His	51	45.10	4.18	−0.03	0.028	0.293	382	4.95	0.089
Ile	57	104.7	7.33	−0.021	0.043	0.629	483	7.75	0.009
Leu	49	160.2	12.67	−0.05	0.056	0.372	440	9.50	0.025
Lys	57	75.52	5.31	0.067	0.028	0.021	439	5.38	0.067
Met	57	25.47	2.51	0.01	0.056	0.849	467	15.28	0.000
Phe	57	46.24	2.83	−0.016	0.017	0.373	382	2.59	0.091
Thr	52	104.4	20.37	0.24	0.28	0.681	657	118.5	0.001
Val	57	218	16.34	−0.09	0.079	0.279	557	14.22	0.025

RPL, rumen-protected lysine; β_0_, overall intercept; SE (β_0_), standard errors of intercept; β_1_, slope for RPL inclusion levels; SE (β_1_), standard error of the slope; AIC, Akaike information of criterion; RMSE, root-means square error; N, number of data; R^2^, coefficient of determination; FCM, fat-corrected milk; ECM, energy-corrected milk; DMI, dry matter intake; EAA, essential amino acids; Arg, arginine; His, histidine; Ile, isoleucine; Leu, leucine; Lys, lysine; Met, methionine; Phe, phenylalanine; Thr, threonine; Val, valine.

**Table 5:** Results of meta-regression analysis of the relationships between RPLM inclusion levels during early lactating dairy cows’ production performance and plasma amino acids concentrations

Response variable	N	Parameter estimates				Model statistics			
	
		β_0_	SE (β_0_)	β_1_	SE (β_1_)	p-value	AIC	RMSE	R^2^
Production performance									
Milk yield (kg/d)	86	33.69	1.214	0.014	0.006	0.028	447	0.885	0.195
3.5% FCM (kg/d)	86	32.87	1.043	0.007	0.009	0.439	459	1.084	0.017
ECM (kg/d)	78	34.44	1.142	0.013	0.01	0.212	418	1.151	0.087
DMI (kg/d)	79	21.68	0.633	−0.002	0.004	0.601	337	0.552	0.022
Milk yield/DMI	79	1.55	0.045	0.001	0.0004	0.019	−8.3	0.064	0.229
ECM/DMI	79	1.60	0.045	0.001	0.0004	0.036	−46	0.057	0.242
Milk fat (%)	86	3.69	0.089	0.0005	0.0008	0.521	78	0.19	0.002
Milk protein (%)	82	3.14	0.055	0.0001	0.0005	0.796	2.2	0.05	0.150
Milk lactose (%)	61	4.80	0.024	−0.0003	0.0002	0.297	−54	0.04	0.003
Milk fat (g/d)	82	1,230	35.6	0.087	0.523	0.569	1,102	59.67	0.000
Milk protein (g/d)	78	1,039	32.41	1.027	0.096	0.001	787	45.77	0.479
Milk lactose (g/d)	57	1,676	99.85	0.249	0.505	0.659	413	48.41	0.024
MUN (mg/dL)	30	13.26	0.754	0.002	0.002	0.425	87	0.27	0.012
Plasma EAA									
Arg	34	79.57	10.76	0.059	0.028	0.585	259	4.16	0.047
His	31	51.56	5.429	−0.013	0.028	0.372	191	3.52	0.031
Ile	34	99.02	10.709	0.006	0.034	0.388	268	4.60	0.000
Leu	26	188.8	25.47	0.007	0.065	0.382	209	8.91	0.033
Lys	34	76.52	8.053	0.096	0.033	0.671	240	5.21	0.229
Met	34	17.92	3.08	0.075	0.025	0.004	204	3.62	0.100
Phe	34	47.30	4.79	−0.004	0.016	0.063	201	2.18	0.058
Thr	25	85.45	10.02	0.063	0.047	0.034	243	3.86	0.003
Val	32	202.8	22.66	−0.044	0.071	0.256	306	9.24	0.000

RPLM, rumen-protected lysine + methionine; N, number of data; β_0_, overall intercept; SE (β_0_), standard errors of intercept; β_1_, slope for RPLM inclusion levels; SE (β_1_), standard error of the slope; AIC, Akaike information of criterion; RMSE, root-means square error; R^2^, coefficient of determination; FCM, fat-corrected milk; ECM, energy-corrected milk; DMI, dry matter intake; EAA, essential amino acids; MUN, milk urea nitrogen; Arg, arginine; His, histidine; Ile, isoleucine; Leu, leucine; Lys, lysine; Met, methionine; Phe, phenylalanine; Thr, threonine; Val, valine.

## SUPPLEMENTARY MATERIAL

**Supplementary Table S1**. Rumen-protected Lys (RPL) and covariates evaluations on the lactational performance of dairy cows

**Supplementary Table S2**. Rumen-protected Lys+Met (RPLM) and covariates evaluations on the lactational performance of dairy cows

**Supplementary Table S3**. Evaluation of models’ candidate based on complete assessment of covariates on milk yield (kg/d)

**Supplementary Table S4**. Groups comparison based on categorical data and their interaction effects between group and diet (top-dress vs deficient diets)
