# Supplementary material for: Relationships between dietary rumen-protected lysine and methionine with the lactational performance of dairy cows — A meta-analysis
Source: Anim Biosci. 2023 Aug 22;36(11):1666–84. doi: 10.5713/ab.23.0084 (PMC10623038; doi:10.5713/ab.23.0084)
Supplement: Supplementary file 1 [file ab-23-0084-Supplementary-Table-1.pdf]

TABLE S1. Rumen-protected Lys (RPL) and covariates evaluations on the lactational performance of dairy cows

Model parameters	Response variables								
	Milk yield, kg/d	FCM, kg/d	ECM, kg/d	DMI	Milk/DMI	ECM/DMI	Milk fat, g/d	Milk protein, g/d	Milk lactose, g/d
β_0	34.77	34.68	35.68	22.07	1.59	1.64	1307	1079	1730
SE(β_0)	1.024	1.068	1.189	0.456	0.045	0.056	44.88	33.09	70.89
β_1	-0.018	-0.007	-0.0056	0.0014	-0.001	-0.0002	0.045	-0.032	-0.898
SE (β_1)	0.009	0.008	0.0079	0.0016	0.0003	0.0003	0.329	0.172	0.565
P-value	0.048	0.372	0.477	0.400	0.033	0.443	0.890	0.852	0.116
β_2	0.983	1.155	1.234	0.013	0.060	0.069	38.06	37.33	57.79
SE (β_2)	0.543	0.536	0.546	0.147	0.023	0.024	21.980	13.080	34.314
P-value	0.862	0.626	0.537	0.739	0.253	0.107	0.662	0.263	0.835
$\beta_1\times\beta_2$	-0.026	-0.023	-0.022	0.0006	-0.001	-0.001	-0.706	-0.528	-1.519
SE ($\beta_1\times\beta_2$)	0.007	0.007	0.007	0.001	0.0003	0.0003	0.286	0.144	0.484
P-value	0.001	0.002	0.002	0.664	0.002	0.003	0.015	0.001	0.003
β_3	3.255	3.848	-0.131	0.252	0.221	-75.4	123.3	391.21	-4.926
SE (β_3)	2.990	3.288	0.981	0.127	0.149	133.3	71.96	229.6	10.34
P-value	0.281	0.246	0.894	0.051	0.144	0.574	0.091	0.097	0.636
$\beta_1\times\beta_3$	-0.25	-0.199	-0.191	0.006	-0.009	-0.007	-6.083	-4.136	-14.94
SE($\beta_1\times\beta_3$)	0.036	0.035	0.034	0.007	0.001	0.001	1.322	0.731	2.517
P-value	<0.001	<0.001	<0.001	0.369	<0.001	<0.001	<0.001	<0.001	<0.001
Early × Mid	<0.001	<0.001	0.001	0.504	<0.001	0.001	0.07	<0.001	<0.001
TD × AAD	0.051	0.229	0.243	0.618	0.041	0.163	0.768	0.208	0.049

β_1 = intercept; β_1 = levels of RPL; β_2 = levels of CP; β_3 = levels of NE_L; SE = standard error; TD = top-dress; DD = deficient diets
