# Supplementary material for: Relationships between dietary rumen-protected lysine and methionine with the lactational performance of dairy cows — A meta-analysis
Source: Anim Biosci. 2023 Aug 22;36(11):1666–84. doi: 10.5713/ab.23.0084 (PMC10623038; doi:10.5713/ab.23.0084)
Supplement: Supplementary file 2 [file ab-23-0084-Supplementary-Table-2.pdf]

TABLE S2. Rumen-protected Lys+Met (RPLM) and covariates evaluations on the lactational performance of dairy cows

Model parameters	Response variables								
	Milk yield, kg/d	FCM, kg/d	ECM, kg/d	DMI	Milk/DMI	ECM/DMI	Milk fat, g/d	Milk protein, g/d	Milk lactose, g/d
β_0	32.94	32.17	33.04	21.67	1.52	1.54	1204	1015	1598
SE(β_0)	1.077	0.941	1.255	0.565	0.039	0.052	33.44	29.62	87.66
β_1	0.012	0.006	0.008	-0.002	0.001	0.001	0.056	0.787	0.443
SE (β_1)	0.005	0.007	0.007	0.003	0.0003	0.0003	0.412	0.215	0.396
P-value	0.059	0.454	0.261	0.468	0.014	0.063	0.793	0.001	0.427
β_2	0.453	0.915	0.843	0.184	0.000	0.03	33.12	9.24	16.83
SE (β_2)	0.155	0.185	0.189	0.089	0.006	0.009	9.285	5.417	10.240
P-value	0.005	<0.001	<0.001	0.065	0.557	0.001	0.061	0.077	0.112
$\beta_1\times \beta_2$	0.002	-0.001	0.0001	-0.002	0.0003	0.0001	-0.129	0.093	-0.031
SE ($\beta_1\times \beta_2$)	0.001	0.0017	0.002	0.001	0.0001	0.0001	0.099	0.051	0.085
P-value	0.174	0.521	0.953	0.04	<0.001	0.161	0.197	0.074	0.719
β_3	5.036	1.479	3.623	-0.627	0.214	0.205	-51.9	86.63	247.2
SE (β_3)	1.607	1.827	2.033	1.169	0.063	0.077	73.77	54.59	153.6
P-value	0.002	0.202	0.083	0.595	0.002	0.012	0.846	0.126	0.132
$\beta_1\times \beta_3$	0.042	0.077	0.078	-0.012	0.003	0.005	4.329	1.799	2.521
SE($\beta_1\times \beta_3$)	0.012	0.016	0.017	0.009	0.0004	0.0006	0.791	0.533	2.534
P-value	0.002	<0.001	<0.001	0.201	<0.001	<0.001	<0.001	0.002	0.338
Early × Mid	0.094	0.725	0.398	0.756	0.015	0.058	0.952	0.001	0.665
TD × AAD	0.285	0.855	0.621	0.883	0.096	0.259	0.988	0.006	0.849

β_1 = intercept; β_1 = levels of RPLM; β_2 = levels of CP; β_3 = levels of NE_L; SE = standard error; TD = top-dress; DD = deficient diets
