# Supplementary material for: Relationships between dietary rumen-protected lysine and methionine with the lactational performance of dairy cows — A meta-analysis
Source: Anim Biosci. 2023 Aug 22;36(11):1666–84. doi: 10.5713/ab.23.0084 (PMC10623038; doi:10.5713/ab.23.0084)
Supplement: Supplementary file 3 [file ab-23-0084-Supplementary-Table-3.pdf]

TABLE S3. Evaluation of models' candidate based on complete assessment of covariates on milk yield (kg/d)

Model	Rumen-protected Lys				Rumen-protected Lys+Met			
parameters	1	2	3	4	5	6	7	8
β_0	35.22	34.97	31.60	34.31	32.94	33.55	33.58	30.66
SE(β_0)	1.024	1.186	1.186	1.781	1.077	1.192	1.281	1.968
β_1	0.001	0.015	0.046	0.011	0.012	0.011	0.001	0.011
SE (β_1)	0.009	0.003	0.003	0.006	0.005	0.005	0.005	0.008
P-value	0.048	<0.001	<0.001	0.022	0.059	0.056	0.000	0.154
β_2			0.607				0.615	
SE (β_2)			0.275				0.223	
P-value			0.031				0.009	
Model performance								
v	1.207	1.030	1.006	1.041	1.044	1.057	1.024	1.016
μ	-0.027	-0.002	-0.029	0.000	0.001	0.000	-0.033	0.001
Cb	0.982	1.000	1.000	0.999	0.999	0.998	0.999	1.000
r	0.980	0.996	0.996	0.997	0.992	0.992	0.993	0.996
CCC	0.962	0.995	0.996	0.997	0.991	0.990	0.992	0.996
MSPE	4.209	0.672	0.608	0.489	0.520	0.843	0.782	0.495
RMSPE	6.043	2.355	2.239	2.034	2.173	2.713	2.614	2.237
AIC	1291	698	655	366	581	446	404	194
R^2	0.000	0.134	0.002	0.276	0.037	0.000	0.000	0.089

Model 1 = RPL using all data; Model 2 = RPL (early lactation data); Model 3 = Model 2 + CP; Model 4 = RPL (early lactation data and top-dress); Model 5 = RPLM using all data; Model 6 = RPLM (early lactation data); Model 7 = Model 6 + CP; Model 8 = RPL (RPLM (early lactation data and top dress)

β_0 = overall intercept; SE (β_0) = standard errors of intercept; β_1 = slope for either RPL or RPLM; SE (β_1) = standard error of the slope; β_2 = slope of dietary CP levels; R^2 = regression coefficient; AIC = Akaike information of criterion; *Cb* = model accuracy; *r* = Pearson

correlation coefficient as a measure of model precision; RMSPE = square root of the mean square prediction error; CCC = concordance correlation coefficient
