# Supplementary material for: Relationships between dietary rumen-protected lysine and methionine with the lactational performance of dairy cows — A meta-analysis
Source: Anim Biosci. 2023 Aug 22;36(11):1666–84. doi: 10.5713/ab.23.0084 (PMC10623038; doi:10.5713/ab.23.0084)
Supplement: Supplementary file 4 [file ab-23-0084-Supplementary-Table-4.pdf]

TABLE S4. Groups comparison based on categorical data and their interaction effects between group and diet (top-dress *vs* deficient diets)

Parameters	Group		SEM	*P*-value		Group		SEM	*P*-value	
	CON	RPL		Group	Group × Diet	CON	RPLM		Group	Group × Diet
Production performance										
Milk yield, kg/d	42.30	42.71	1.293	0.812	0.925	32.51	32.80	0.845	0.793	0.998
3.5% FCM, kg/d	43.27	44.03	1.262	0.651	0.767	30.69	30.68	0.980	0.993	0.802
ECM, kg/d	43.09	43.83	1.204	0.642	0.782	31.46	31.63	1.574	0.916	0.834
DMI, kg/d	22.25	22.64	1.422	0.492	0.428	21.01	20.72	0.673	0.669	0.885
Milk yield/DMI	1.59	1.58	0.113	0.781	0.481	1.52	1.57	0.045	0.349	0.967
ECM/DMI	1.41	1.41	0.153	0.998	0.453	1.49	1.52	0.054	0.705	0.817
Milk fat, %	3.11	3.14	0.174	0.739	0.485	3.44	3.5	0.111	0.579	0.596
Milk protein, %	2.62	2.61	0.109	0.859	0.700	3.13	3.15	0.042	0.649	0.896
Milk lactose, %	4.55	4.56	0.049	0.819	0.854	4.84	4.81	0.034	0.243	0.923
Milk fat, g/d	1560	1591	46.94	0.613	0.631	1189	1181	29.41	0.850	0.907
Milk protein, g/d	1236	1247	67.53	0.817	0.818	1001	1028	23.62	0.409	0.495
Milk lactose, g/d	1658	1695	104.60	0.628	0.968	1594	1545	62.28	0.588	0.903
N Metabolism										
N Intake, g/d	641.5	691.5	16.04	0.998	0.248	576	572	17.31	0.883	0.346
N urine, g/d	220.5	219	13.22	0.839	0.978	175	180	30.14	0.894	0.524
N fecal, g/d	195.5	195.5	14.37	0.993	0.327	200	184	21.54	0.549	0.886
N Milk, g/d	178.5	181	29.36	0.896	0.820	140	142	6.390	0.814	0.728
N balance, g/d	53.25	50.5	21.38	0.783	-	54.69	53.29	10.83	0.916	0.623
Plasma EAA										
Arg	115	116	4.835	0.525	0.816	92.98	96.8	9.463	0.764	0.853
His	97.17	97.84	13.15	0.892	0.981	52.46	49.34	4.885	0.558	0.563
Ile	73	72	5.445	0.788	0.582	106.2	106.4	10.67	0.985	0.368

Leu	149.5	152	10.28	0.447	0.439	230.3	245.3	21.98	0.482	0.314
Lys	117	120	8.456	0.286	0.561	67.38	68.34	33.54	0.977	0.123
Met	28.05	27.57	1.578	0.818	0.853	19.68	27.33	2.262	0.005	0.849
Phe	88.82	88.69	5.248	0.944	0.683	45.28	43.84	4.680	0.766	0.367
Thr	10.66	22.56	64.88	0.629	0.813	90.49	90.87	6.038	0.963	0.389
Val	302	308	26.76	0.534	0.514	239	245	26.62	0.800	0.369
